# ln*Ce*DB: Database of Human Long Noncoding RNA Acting as Competing Endogenous RNA

**DOI:** 10.1371/journal.pone.0098965

**Published:** 2014-06-13

**Authors:** Shaoli Das, Suman Ghosal, Rituparno Sen, Jayprokas Chakrabarti

**Affiliations:** 1 Computational Biology Group, Indian Association for the Cultivation of Science, Kolkata, West Bengal, India; 2 Gyanxet, Kolkata, West Bengal, India; Telethon Institute of Genetics and Medicine, Italy

## Abstract

Long noncoding RNA (lncRNA) influences post-transcriptional regulation by interfering with the microRNA (miRNA) pathways, acting as competing endogenous RNA (ceRNA). These lncRNAs have miRNA responsive elements (MRE) in them, and control endogenous miRNAs available for binding with their target mRNAs, thus reducing the repression of these mRNAs. **ln** ***Ce*** **DB** provides a database of human lncRNAs (from GENCODE 19 version) that can potentially act as ceRNAs. The putative mRNA targets of human miRNAs and the targets mapped to AGO clipped regions are collected from TargetScan and StarBase respectively. The lncRNA targets of human miRNAs (up to GENCODE 11) are downloaded from miRCode database. miRNA targets on the rest of the GENCODE 19 lncRNAs are predicted by our algorithm for finding seed-matched target sites. These putative miRNA-lncRNA interactions are mapped to the Ago interacting regions within lncRNAs. To find out the likelihood of an lncRNA-mRNA pair for actually being ceRNA we take recourse to two methods. First, a ceRNA score is calculated from the ratio of the number of shared MREs between the pair with the total number of MREs of the individual candidate gene. Second, the P-value for each ceRNA pair is determined by hypergeometric test using the number of shared miRNAs between the ceRNA pair against the number of miRNAs interacting with the individual RNAs. Typically, in a pair of RNAs being targeted by common miRNA(s), there should be a correlation of expression so that the increase in level of one ceRNA results in the increased level of the other ceRNA. Near-equimolar concentration of the competing RNAs is associated with more profound ceRNA effect. In ln*Ce*DB one can not only browse for lncRNA-mRNA pairs having common targeting miRNAs, but also compare the expression of the pair in 22 human tissues to estimate the chances of the pair for actually being ceRNAs. **Availability**: Downloadable freely from http://gyanxet-beta.com/lncedb/.

## Introduction

A major part of the transcriptome of higher eukaryote does not code for any protein, but functions as regulatory RNAs. Long noncoding RNAs (lncRNAs) are long transcripts (generally more than 200 bases) that in many ways are similar to protein coding transcripts, except for the lack of a meaningful coding sequence (CDS) or open reading frame (ORF) [1]–[2]. In the last decade it has become possible to annotate a greater number of lncRNA transcripts. Consequently the study of them has gained much significance as many of them have been linked with epigenetic, transcriptional and post-transcriptional regulation of gene expression [3]. Despite having generally a lower level of concentration than protein coding transcripts, lncRNAs exhibit more tissue specific expressions [4]–[5]. A vast set of lncRNA transcripts are differentially expressed during development where many of them play critical roles [6]–[9]. LncRNAs are now known to have a major involvement in cancer [10]–[11]. Though, till now, a majority of the lncRNAs have been linked with epigenetic modulation of gene expressions [12]–[13], they can also regulate gene expression by transcriptional or post transcriptional modes [14]–[15]. LncRNAs can influence post-transcriptional regulation by interfering with the miRNA pathways, by acting as competing endogenous RNAs (ceRNAs) [16]. In recent years it has been discovered that endogenous RNAs (mRNAs, pseudogenes, long noncoding RNAs or circular RNAs) compete with each other for the limited pool of cellular microRNAs (miRNAs) and thus affect the competing RNA's level. These RNAs have miRNA responsive elements (MRE), i.e., the miRNA binding sites in them, and act as miRNA sponges to control endogenous miRNAs available for binding with their target mRNAs, thus reducing the repression of these mRNAs [17]–[24]. This phenomenon adds a significant new dimension to the miRNA mediated regulation of gene expression in cells. ceRNAs are important regulators in cell cycle control and tumor suppression (e.g. PTEN-P1 blocking miR-19b and miR-20a from binding to PTEN tumor suppressor [17]–[19]), modulating self-regulation in hepatocellular carcinoma (HCC) (HULC lncRNA acts as ceRNA of the protein coding gene PRKACB that induces activation of CREB which in turn is involved in upregulation of HULC [20]) as well as in developmental stages (e.g. linc-MD1 blocking miR-133 from binding to transcription factors involved in myogenic differentiation [21] and H19 blocking the miRNA let-7 to affect muscle differentiation in vitro [22]). Circular RNAs have recently been shown to be involved in pathways of cancer and many other diseases [23], [24].

Due to the availability of huge lncRNA datasets from recent GENCODE versions [25], 13870 lncRNA genes in GENCODE 19, it has become imperative to uncover the potential functions of these transcripts. In the light of new findings on ceRNAs and lncRNA-miRNA interactions, we developed a database, ln*Ce*DB, of human lncRNAs that can potentially act as ceRNAs (based on the newly available GENCODE 19 annotated lncRNAs). Recently, databases describing lncRNA-miRNA interactions, like miRCode [26], Diana-lncBase [27], lncRNome [28] and StarBase v2.0 [29], have become available. But none of them documents miRNA interactions with lncRNAs annotated past GENCODE 17. We used lncRNA-mRNA interaction pairs from miRCode database of miRNA targets for lncRNAs in GENCODE 11 [26], and for the newly enlisted lncRNAs in GENCODE 19, we predicted seed-matched miRNA targets using our algorithm. We mapped these putative miRNA-lncRNA interactions into the Ago-interacting regions within lncRNAs, collected from a recent study [30]. In ln*Ce*DB, the users can also browse for miRNA targets on recently available GENCODE 19 lncRNAs not available from other databases. Moreover, the objective of lnCeDB is not just describing lncRNA-miRNA interactions, but providing researchers with a database of human lncRNAs that can potentially act as ceRNAs to protein coding genes. The chances of an lncRNA-mRNA pair for actually being ceRNA depend not only on the fact that they are targeted by common miRNA(s), but also other factors like relative concentrations of individual component ceRNAs and the number of shared MREs. ln*Ce*DB is built by taking into consideration these varied and complex features.

A previously published database of ceRNAs, ceRDB, provides data of mRNAs that can putatively act as ceRNAs, but it does not have information about lncRNAs [31]. It should also be noted that unlike the ceRDB database, that used putative miRNA-mRNA interactions predicted by TargetScan [32], we include mRNA-miRNA and miRNA-lncRNA interactions predicted from AGO CLIP-Seq data [30], [33]. The user can limit the target search within regions of AGO interaction, significantly reducing false-positive target. Another recently published database, StarBase v2.0 include ceRNA pairs predicted from available AGO PAR-CLIP datasets [29]. However, the use of only the PAR-CLIP data for prediction of ceRNA pairs limits the result set to only a few cell lines where the AGO PAR-CLIP was performed. As mentioned earlier, our dataset includes, but is not limited to predictions from just the AGO CLIP-SEQ data. This gives the user a broader set of probable ceRNA pairs in many human tissues, and also the option to narrow down the search to only the AGO interacting regions as available from AGO CLIP-SEQ data.

As mentioned earlier, the chances of an lncRNA-mRNA pair actually being ceRNA depend not just on the fact that they are targeted by common miRNA(s), but also on the number of miRNA responsive elements (MRE), number of distinct miRNAs targeting both transcripts and the concentration and cellular levels of the competing RNAs. A study by Ala et al provides a comprehensive view on the ceRNA network and the possible outcome of perturbation in the components (miRNA and competing RNA levels) of the network [34]. The authors showed that the relative expressions of competing RNAs play a vital part in determining the ceRNA effect. While, for a pair of ceRNAs, it is seen that the competing RNA with higher expression has greater ceRNA effect on the other competing RNA, it has also been observed that competing RNAs with near-equal expression exhibit more robust ceRNA effect than other ceRNA pairs having largely different expressions [34]. Thus, the information on the concentration levels of the two RNAs making the ceRNA pair is very crucial. Also, to determine the potential cross-regulation of a ceRNA pair, it is very important to check the co-expression of shared miRNAs along with the ceRNA pair. One major drawback of the existing ceRNA databases, other than ln*Ce*DB, is that they do not offer the option to the users to check the co-expression of the ceRNA pair and the shared miRNAs. Following from the observation by Ala et al, to estimate the chances of an lncRNA-mRNA pair for actually being ceRNAs in particular tissues, ln*Ce*DB offers users the possibility to browse for lncRNA-mRNA pairs targeted by common miRNAs (sorted by the number of shared miRNAs of the pair) and compare the expression of the pair in 22 human tissues (from RNA-Seq expression data from Cabili et al [35]). Moreover, ln*Ce*DB also provides users with the information on the shared miRNAs co-expressed in each of the 22 different tissues. This feature is not offered by any other ceRNA database.

To assess the likelihood of an lncRNA-mRNA pair to act as ceRNAs, we provide two different methods. In the first approach, we calculate the P-value for each ceRNA pair by hypergeometric test, similar to the study by Sumazin et al [19] and StarBase v2.0 [29], considering the number of shared miRNAs between the pair against the total number of miRNAs targeting each component in the pair, i.e. the lncRNA and the mRNA. But in the second approach, unlike other ceRNA databases that predict the likelihood for being ceRNAs by the number of shared miRNAs [19], [29], we calculate a ceRNA score for each probable ceRNA pair by taking into consideration the number of shared MREs against the total number of MREs for the candidate lncRNA. A major drawback of the other ceRNA databases [29] is that they calculate the likelihood of a pair of genes to act as ceRNA by considering only the number of shared miRNAs between the pair. But there is a certain importance to the number of shared MREs compared to the number of shared miRNAs between the ceRNA pair. Thus, a candidate lncRNA having 100 MREs for 1 shared miRNA would be considered lesser than a candidate lncRNA with 2 MREs for 2 shared miRNAs in some of the existing databases. We believe that the number of shared MREs would be more appropriate instead of the number of shared miRNAs between the ceRNA pair. And that makes ln*Ce*DB different from the existing databases on ceRNA. In ln*Ce*DB, the ceRNA score, along with the provision for checking relative expressions of the ceRNA pair over different tissues, offer users a better assessment of the potential ceRNAs. We believe, therefore, that ln*Ce*DB addresses the finer aspects of post transcriptional gene expression regulation in human.

## Results

**Figure 1:**
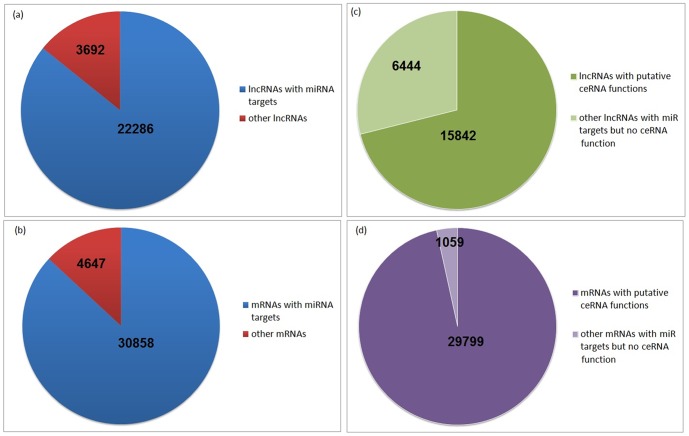
A statistics of the database contents in ln*Ce*DB. (a) Fraction of GENCODE 19 lncRNA transcripts with putative miRNA targets. (b) Fraction of mRNAs with predicted miRNA targets. (c) Fraction of lncRNAs with predicted ceRNA function compared to all lncRNAs with putative miRNA targets. (d) Fraction of mRNAs with predicted ceRNA function compared to all mRNAs with putative miRNA targets.

ln*Ce*DB contains dataset for human genome wide lncRNAs that potentially can act as ceRNAs. Presently we have miRNA targets on 22286 lncRNAs out of 25978 lncRNA transcripts in GENCODE 19. miRNA targets for lncRNAs in GENCODE 11 were collected from miRCode database and targets for newly enlisted lncRNAs in GENCODE 19 were predicted by our algorithm (see section 2). Among the 22286 lncRNAs with putative miRNA target sites, 15842 lncRNAs were predicted to be working as potential ceRNAs. Figure 1 shows a statistics of the database contents in lnCeDB.

### lncRNA-miRNA interactions mapping into Ago interaction sites within lncRNA loci

Among the putative lncRNA-miRNA interactions, 72 interaction pairs were mapped to the Ago-interaction sites within lncRNA transcript loci which contained 41 miRNAs targeting 27 lncRNA transcripts. ceRNAs specifically corresponding to these PAR-CLIP supported miRNA target interactions are browsable in ln*Ce*DB.

### Putative mRNA-miRNA interactions from TargetScan

Conserved human miRNA targets on mRNAs were collected from TargetScan [32]. The current version stores miRNA targets on 30858 mRNA transcripts for human.

### mRNA-miRNA interactions derived from Ago interacting regions within mRNA

miRNA targets on 6090 mRNAs were collected from StarBase; these targets were predicted from CLIP-Seq data by five most widely used miRNA target prediction tools (see section 2).

### Expression data for a ceRNA pair over 22 tissues

Users can compare the cellular levels of the competing RNAs to estimate the chances of the pair for actually being ceRNAs by viewing the tissue specific expression of a ceRNA pair over 22 human tissues. Presently we have tissue specific expressions for 7017 lncRNA genes and 17701 protein coding genes.

### Experimentally verified lncRNAs acting as ceRNAs

A small number of lncRNAs are reported and experimentally verified as ceRNAs. Experimentally validated lncRNA ceRNAs are identified in ln*Ce*DB. The reported lncRNA-ceRNAs include lncRNA HULC acting as ceRNA of the gene PRKACB [20], lincRNA MD1 working as ceRNA of the gene MAML1 [21] and linc-RoR acting as ceRNA of the pluripotency associated transcription factors SOX2 and NANOG [36]. Table 1 lists the experimentally verified lncRNA ceRNAs in ln*Ce*DB.

**Table 1:** Experimentally verified lncRNA ceRNAs in ln*Ce*DB.

lncRNA acting as ceRNA	Competing protein coding gene	Shared miRNA	ceRNA score	Reference
HULC (Highly Upregulated in Liver Cancer)	PRKACB	miR-372	0.026 (p-value = 0.001)	[20]
lincRNA MD1	MAML1	miR-133	0.022 (p-value = 0.02)	[21]
H19	Targets of hsa-let-7	Let-7	-	[22]
Linc-RoR (Regulator of Reprogramming)	SOX2 and NANOG	miR-145	0.038 (p-value = 0.008)	[36]
PTCSC3	Targets of miR-574-5p in thyroid cancer cell line	miR-574-5p	-	[37]

### Utility

**Figure 2:**
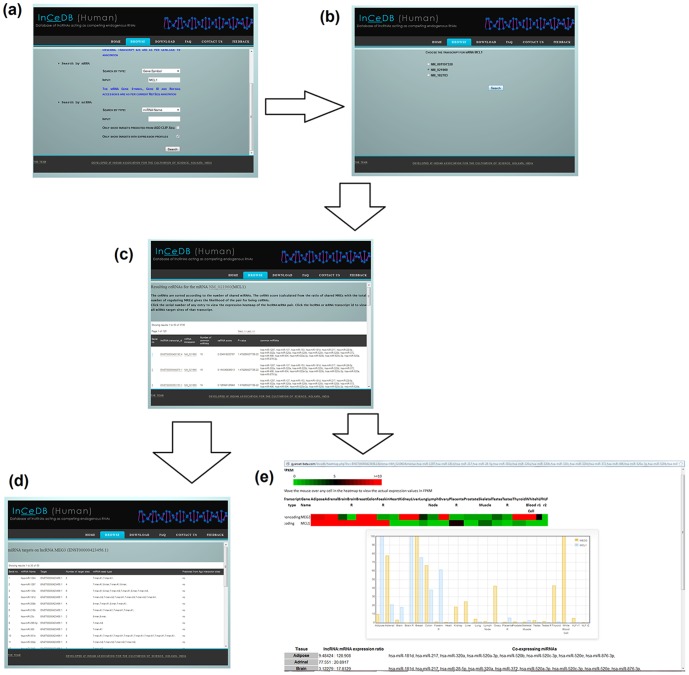
The flow diagram for browsing ceRNAs in ln*Ce*DB. (a) lnCeDB is searched by the gene symbol MCL1. (b) An intermediate page shows the different transcripts of the gene MCL1. (c) Upon choosing a transcript, the results page shows the potential lncRNAs working as ceRNA for the chosen transcript of MCL1. (d) By clicking on a lncRNA or mRNA id in the ceRNA table, the user views all miRNA targets on the chosen transcript. (e) By clicking on a serial number in the ceRNA table, the user views the expression heatmap and bar chart for the ceRNA pair along with co-expressed shared miRNAs in 22 human tissues.

Users can browse for ceRNA candidates for a protein coding gene (by gene symbol, gene id or refseq accession) and/or lncRNA gene (by gene name, ensemble gene id or ensemble transcript id) and/or miRNA name. lncRNA-mRNA pairs targeted by common miRNAs are displayed as potential ceRNAs. The list of ceRNAs is sorted by the number of shared miRNAs. The miRNA targets for a particular lncRNA or mRNA can also be browsed in our database by choosing a particular lncRNA/mRNA from the ceRNA list. By choosing a particular record for a ceRNA pair, users can view the RNA-Seq expressions of the lncRNA-mRNA pair over 22 human tissues in form of a heat map (if the expression data for the corresponding lncRNA and mRNA is available) and a bar chart with actual FPKM values appearing in tool tip text. The users can also view the co-expressed miRNAs shared between the ceRNA pair in each of the 22 tissues. Figure 2 shows a flow diagram for the usability of lnCeDB.

## Discussion

As competing endogenous RNAs are crucial new determinants of gene expression regulation [38], new data sources are needed. Following the availability of huge dataset of annotated lncRNA transcripts from GENCODE project, the possible functions of the transcripts have to be addressed. In ln*Ce*DB, we have tried to explore the potential sponge activity of recently annotated lncRNAs in the miRNA mediated gene regulation networks at human genome wide scale. Unlike other ceRNA databases, ln*Ce*DB includes but is not limited to miRNA targets on protein coding and lncRNA transcripts predicted from Ago interaction sites within them. The advantage is that it reduces the false positive target detection in our miRNA-target interaction dataset and enhances the reliability of the prediction. At the same time, the dataset is not limited to predictions from only a few cell lines where AGO PAR-CLIP were performed. Also, in ln*Ce*DB we considered, for the first time, that ceRNA activity largely depends on the relative concentration of the components of a ceRNA network, i.e. the pair of competing RNAs and also the miRNAs they compete for. The provision for checking the tissue specific expression for a potential ceRNA pair (whenever available) along with the co-expression of shared miRNAs, gives the user a higher chance of identifying the most likely ceRNA candidates in a tissue of interest.

One interesting example of a putative ceRNA pair identified by ln*Ce*DB is the lncRNA maternally expressed 3 (MEG3) and a transcript (the longer isoform) of the protein coding gene Myeloid Cell Leukemia Sequence 1 (MCL1). MEG3 is a maternally expressed imprinted gene encoding a number of alternatively spliced lncRNA transcripts. It interacts with the tumour suppressor P53, and is supposedly a tumour suppressor itself. MEG3 is expressed in many normal tissues including breast, colon, liver, ovary but its expression is lost in many tumour cells. Interestingly, it has been reported that MEG3 is targeted by miRNAs [39]. MCL1 is a member of the BCL2 family and it has three isoforms. The longer isoform is anti-apoptotic whereas the shorter isoforms are pro-apoptotic. The ceRNA pair MEG3-MCL1 putatively shares 16 common miRNAs including miR-28, miR-181d, miR-520a, miR-520b and miR-876-3p and show comparable high co-expressions in breast and colon, especially in colon (MEG3 (66.2): MCL1 (37.9)). The co-expression pattern of MEG3 and MCL1, along with the co-expressed shared miRNAs indicates that MEG3 may act as a ceRNA to MCL1 in colon. Interestingly, the MEG3 gene locus has been reported to be hypermethylated in colorectal cancer cells [40] indicating the possible perturbation of MEG3 lncRNA expression in colorectal carcinoma. Furthermore, in a colorectal cancer cell, the anti-apoptotic MCL1 has been reported to be regulated (in vitro) by a number of miRNAs, including miR-876-3p [41] which we predicted to be shared by MEG3 and MCL1. Together, these observations suggest that there may be a disruption of the potential MEG3-MCL1 ceRNA network in colorectal cancer cells as opposed to the normal colon cells. This observation, however, needs to be validated by further investigations. This example shows the importance of ln*Ce*DB over other ceRNA databases as no other ceRNA database allows the users to check the co-expression patterns of the competing RNAs and the shared miRNAs in different tissue types. Some other interesting observations from ln*Ce*DB are MEG3 and CMPK1 as potential ceRNA pair with near-equal expression signature in colon and ovary and MALAT1-PRKACB potential ceRNA pair in liver.

We believe this database will help researchers in deciphering the larger and more complex scenario of miRNA mediated gene regulatory networks in human in the real world of ceRNAs.

## Materials and Methods

### Data Source

We collected predicted miRNA target sites on human mRNA transcripts from TargetScan. The miRNA-mRNA interactions mapped to the AGO interacting regions were collected from StarBase database [33]. StarBase houses datasets of mRNA-miRNA interactions predicted from AGO CLIP-Seq data (AGO interacting regions) by widely used miRNA target prediction tools like TargetScan [32], PITA, PicTar, RNA22 and miRanda. The lncRNA-miRNA interaction dataset (for lncRNAs in GENCODE 11 version) was downloaded from miRCode database [26]. For newly enlisted lncRNAs in GENCODE 19 version, we used our miRNA-target prediction algorithm for finding seed matched miRNA target sites on lncRNAs. The dataset for Ago interacting regions within lncRNA transcript loci was collected from Jalali et al [30]. Tissue specific expressions (RNA-Seq) for lncRNAs and mRNAs in 22 human tissues were used from the dataset of Cabili et al [35]. Tissue specific miRNA expressions (QRT-PCR) were collected from miRNA body-map [42].

### Prediction of miRNA target sites on lncRNAs

We used a 25 base window on the target to run a modified version of the Smith-Waterman alignment (as used in the miRanda algorithm [43]) to find complementary alignment of the 25 base target region with the miRNA. For reducing runtime, at the first step we searched for 6-mer seed region (position 2-7 on miRNA) complementarities on the target. If a match was found, then a 25 base window around the seed-matched site was used for further alignment. For prediction of different types of miRNA target sites (6-mer, 7-mer, 7-merA1 and 8-mer), we changed the seed region definition in each case [43]. We considered transcripts with perfect seed complementarity as well as one base mismatch tolerance (in position 2- 8 or 2-7) in the seed region with 3′ compensatory complementarity (at position 13-18).

### Consideration for target site conservation

We treated conserved and non-conserved target sites separately. We searched for target region conserved among human, chimp, mouse, rat and dog. Genome wide conservation data generated using multiz 46-way alignment (for 46 vertebrate species) was downloaded from UCSC genome browser [44]. Genomic regions (within human genome) conserved within human, chimp, mouse, rat and dog were then extracted and mapped within the coordinates of human mRNAs (downloaded from UCSC genome browser) to get the location of the conserved regions within human mRNAs. Conserved regions of length 8 bases or more were only considered.

### The ceRNA score

We generated the ceRNA score of an lncRNA-mRNA pair targeted by common miRNAs to measure the likelihood of a lncRNA to act as ceRNA to a protein coding gene. The ceRNA score was calculated keeping in mind the observation by Ala et al [34] that genes that shared a large number of distinct miRNAs, as opposed to genes targeted by a small number of shared miRNAs, compared to the total number of miRNAs targeting the individual genes, exhibit more profound ceRNA 
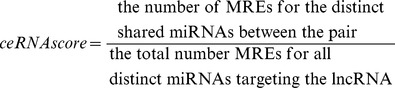
(1)


### Calculating the probability of ceRNA pair to cross-regulate each other

We implemented another measure to assess the likelihood of a ceRNA pair to regulate each other via shared miRNAs. This approach was similar to what has been used in the study of Sumazin et al [19] and in StarBase v2.0 [29]. We calculated the p-value for each potential ceRNA pair by hypergeometric test considering the number of shared miRNAs between a ceRNA pair against the number of miRNAs targeting individual components of the ceRNA pair. The p-value was measured as:
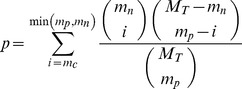
(2)Where,

*M_T_* = Total number of miRNAs in the human genome

*m_p_* = Number of miRNAs interacting with the mRNA (protein-coding)

*m_n_* = Total number of miRNAs interacting with the lncRNA (non protein-coding)

*m_c_* = Number of miRNAs shared between the ceRNA pair

### Implementation

The miRNA target finding algorithm was implemented in JAVA and the web interface of the database ln*Ce*DB was developed using PHP-mySql.
